# PHIRI Rapid Exchange Forum (REF): A key tool for cross-country exchange in times of crisis: Claudia Habl

**DOI:** 10.1093/eurpub/ckac129.646

**Published:** 2022-10-25

**Authors:** C Habl, C Pries

**Affiliations:** International Affairs and Consulting, Gesundheit Österreich GmbH - Austrian National Public Health Institut, Vienna, Austria

## Abstract

The COVID-19 pandemic demanded a quick exchange between experts and institutions supporting national governments in the crisis response literally from the first day onward to provide evidence-based information. There was no such regular cross-country forum established in the field of population health in the beginning of the crisis. Thus, devoted members of the Joint Action on Health Information started already in April 2020 to meet regularly online to foster cross-country exchange. In these meetings, partners could approach each other for questions and shared views in a trusted environment. The European Commission recognized this achievement and supported this exchange as part of the Population Health Research Infrastructure (www.phiri.eu), allowing a quick exchange of data, indicators, good practices and experiences in the COVID-19 crisis response in an efficient manner. Till May 2022 34 REF meetings have taken place, involving project partners, national advisors to ministers/heads of agency, representatives of expert networks (e.g. Healthy Cloud), EU services (ECDC, JRC) and different health stakeholders (e.g. ELIXIR). The bi-weekly 1 hour online meetings cover in a moderated, structured format pre-agreed COVID-19-topics that are proposed by the participating countries and chosen via a survey ex-ante to each meeting). Responses by the countries (backed up by national reports, andguidelines that are briefly presented in the meetings) are compiled and shared immediately after the meeting via the Corona-Corner of the Health Information Portal (www.healthinformationportal.eu/rapid-exchange-forum). Topics covered reach from a discussion on suitable non-pharmaceutical interventions and their application especially prior to the availability of vaccines to child vaccination strategies and overall testing regimes to the most suitable communication tools. In average, 25 to 30 delegates from different, mainly EU member states attend each exchange.

**Key messages:**

• The PHIRI Rapid Exchange Forum demonstrated its importance for regular decision making in the field of population health not only in times of crisis.

• The Rapid Exchange Forum is an integral part of the European Health Information Portal (www.healthinformationportal.eu) which shall be transformed into a sustainable infrastructure.

